# The Effect of an Adding Histidine on Biological Activity and Stability of Pc-pis from *Pseudosciaena crocea*

**DOI:** 10.1371/journal.pone.0083268

**Published:** 2013-12-13

**Authors:** Yong Mao, Sufang Niu, Xin Xu, Jun Wang, Yongquan Su, Yang Wu, Shengping Zhong

**Affiliations:** College of Ocean and Earth Sciences, Xiamen University, Xiamen, Fujian, China; University Hospital Schleswig-Holstein, Campus Kiel, Germany

## Abstract

Pc-pis is a novel piscidin-like antimicrobial polypeptide that was identified in *Pseudosciaena crocea*. Although active against most bacteria tested, Pc-pis was inactive against *Aeromonas hydrophila* and *Pseudomonas aeruginosa*. The Pc-pis analogue Pc-pis-His was designed by adding a histidine residue at the carboxyl terminal. Pc-pis-His demonstrated a more broad-spectrum and stronger antimicrobial activity against a representative set of microorganisms and more potent antiparasitic activity against *Cryptocaryon irritans* trophonts than Pc-pis. The stability assay revealed that Pc-pis-His was active against *Staphylococcus aureus* not only in acidic (pH 5.5–7.3) and relatively low concentration monovalent cation (0–160 mM NaCl) environments but also in alkaline (pH 7.5–9.5), divalent cation (1.25–160 mM MgCl_2_ and 1.25–40 mM CaCl_2_) and high concentration monovalent cation (320–2560 mM NaCl) environments, which indicates that the added histidine residue conferred better salt-, acid- and alkali-tolerance to Pc-pis-His. Pc-pis-His also possessed the desired heat-tolerance, which was reflected by the antimicrobial activity of the peptide after being boiled for 10–60 minutes. Hemolytic activity analysis revealed that Pc-pis-His at concentrations up to 6 µM exhibited no hemolysis against human erythrocytes, with 6 µM being a concentration that is highly active against most of the microorganisms tested, although the hemolytic activity of Pc-pis-His was enhanced compared to Pc-pis. These results provide a unique, reasonable basis for designing novel piscidins with potent, broad-spectrum and stable antimicrobial activity and new insight into the future development of piscidins as potential therapeutic agents against microbial and external protozoan parasite infections.

## Introduction

With the development of marine aquaculture, various infectious diseases can inflict significant economic losses on the aquaculture industry, such as marine cryptocaryonosis (white spot disease), which results in as much as 75% and 50% mortality for *Pseudosciaena crocea* and juvenile *Epinephelus tauvina*, respectively [1], [2]. However, there are still no effective and environmentally friendly therapeutic drugs to control these infectious diseases. Antimicrobial polypeptides (AMPs), existing in almost all living organisms, are crucial components of the natural innate (nonspecific) immunity system in fish and have potent, broad-spectrum antimicrobial and antiparasitic activity in vitro [3]–[5]. There has been increased research in these peptides to identify a new class of antibiotic agents with potential clinical value. However, some AMPs have irrevocable disadvantages, such as weak antimicrobial activity or high hemolytic activity, which limit their therapeutic use [6], [7]. Some other peptides have the potential to be improved in antimicrobial activity, antiparasitic activity or other respects [1], [7], [8]. Therefore, it is very important to design new AMPs that exhibit broader applications, based on the overall structure of the AMPs isolated. Currently, designing new AMPs has become one of the focuses in the field of biology [9]–[12]. In previous studies, some AMPs, such as Pis-1, IsCT and HP (2–20) analogues, exhibited increased desirable biological activity after the incorporation of some amino acids into the various AMPs [10], [11],[13]. These studies demonstrate that the creation of AMP analogues with an extensive use range is a hopeful strategy for the development of potential therapeutic drugs.

To date, there have been many AMPs isolated from teleosts [1], [6]–[8], [14], [15]. Among the most common and potent AMP family present in fish is the piscidins, linear cationic α-helical peptides, except for Atlantic Cod piscidin [3], [7], [16]. We have recently reported a novel piscidin-like antimicrobial cationic peptide, Pc-pis, isolated from *P. crocea* [1]. This peptide is an excellent inhibitor of parasite and most bacterial growth [1]. However, the antimicrobial activity of Pc-pis is selective for the bacteria tested and has no effect on *Aeromonas hydrophila* at the concentration tested (≤96 µM) [1]. *A. hydrophila*, a highly pathogenic bacterium, can cause systemic infections in aquatic animals and considerable economic losses to the aquaculture industry [17], [18]. Thus, it is of great significance to design a new analogue with more broad-spectrum and effective antimicrobial activity based on Pc-pis, which would make the peptide more applicable.

The amino acid histidine, a common basic residue and aromatic residue [19]–[21], is rich in piscidins [1], [6], [7]. For instance, *Epinephelus coioides* epinecidin-1 contains 4 histidine residues (16%) located at position 4, 11, 17 and 25 (carboxyl terminal) [22]. Structurally, the high content of histidine residues confers several important features to piscidins, such as positive charge and electrostatic cation-π interaction, which are crucial for the biological properties of piscidins [6], [23]. In some AMPs, the substitution of glycine with histidine increases the antimicrobial activity of the peptides [6], [10]. Therefore, introducing a histidine residue in Pc-pis may be a good strategy to change the biological properties of the peptide, including an increase in antimicrobial activity.

In this experiment, a new AMP, Pc-pis-His, was designed by adding a histidine residue at the carboxyl terminal of the parent analogue Pc-pis. The study firstly comparatively analyzed the structure biology characteristics of Pc-pis-His and Pc-pis. Then, their activity in a representative set of microorganisms and marine parasite *Cryptocaryon irritans* trophonts and their toxicity to human erythrocytes was evaluated and compared. Furthermore, the salt-, acid-, alkali- and heat-tolerance of Pc-pis-His and Pc-pis were investigated in identical environments. The data provides new insights into the effect of a histidine residue on the structure-antimicrobial activity relationships of piscidins and contributes to the molecule design of potent therapeutic drugs with wide application range based on piscidins.

## Materials and Methods

### 2.1 The design and molecular characterization of the peptide

Based on the amino acid sequence and structure of Pc-pis (IWGLIAHGVGHVGRLIHGLIRG) [1], Pc-pis-His (IWGLIAHGVGHVGRLIHGLIRGH) was designed by adding a histidine residue at the carboxyl terminal. To probe the contribution of the histidine residue to structure, the structure biology characteristics of Pc-pis-His and Pc-pis were comparatively analyzed. The Schiffer-Edmundson helical wheel diagram was obtained using Helical Wheel Projection software (http://rzlab.ucr.edu/scripts/wheel/wheel.cgi). The α-helicity content was predicted using the PredictProtein tool (http://www.predictprotein.org/). The isoelectric point (pI) and the grand average of hydropathicity index were predicted using the ProtParam tool at ExPASy. The charge was calculated by the online tool Peptide Property Calculator (https://www.genscript.com/ssl-bin/site2/peptide_calculation.cgi). The putative membrane-binding affinity of Pc-pis and Pc-pis-His was estimated based on the whole-residue interfacial hydrophobicity of amino acids calculated by Wimley & White [24], [25].

### 2.2 Peptide synthesis and dilution

In this paper, Pc-pis and its analogue Pc-pis-His, with a purity of >98%, were generated at Shanghai Sangon Biological Engineering Technology and Services Co., Ltd. (China) using the solid reaction method. The synthesized peptides were stored desiccated at −20°C until reconstitution in 0.9% normal saline, sterile deionized water or in a 0.01% HAc solution containing 0.2% BSA in sterile deionized water (diluent I), and 2-fold serial dilutions of the reconstituted peptides at final concentrations ranging from 0.375 to 48 µM were used to evaluate the effects of Pc-pis and Pc-pis-His on human erythrocytes, bacteria and *C. irritans*.

### 2.3 Bacterial strains and culture conditions

The gram-positive and gram-negative bacterial strains (gift from Fisheries college, Jimei University, Xiamen, China), filamentous fungi (gift from State Key Laboratory of Marine Environment Science, College of Ocean and Earth Sciences, Xiamen University, Xiamen, China) and yeast (gift from State Key Laboratory of Marine Environment Science, College of Ocean and Earth Sciences, Xiamen University, Xiamen, China) that were used in this study are listed in Table 1. The non-marine and marine bacteria were grown, respectively, in Mueller-Hinton broth/agar medium (MHB/MHA) and saline peptone water (3% Tryptone and 3% NaCl, w/v) at 37°C (mammalian pathogens) or 28°C. The yeast were cultured in YPG (1% yeast extract, 1% peptone, 2% glucose and 0 or 1.5% agar), and the filamentous fungi were cultured in half strength potato dextrose broth/agar (PDB/PDA). The culture temperature of yeast and filamentous fungi both were 28°C.

**Table 1:** Antimicrobial activity of Pc-pis-His and Pc-pis.

Microorganisms	Pc-pis-His		Pc-pis	
	MIC (µM)	MBC (µM)	MIC (µM)	MBC (µM)
Gram-positive bacteria				
*Bacillus Subtilis*	1.5–3	1.5–3	0.75–1.5^a^	1.5–3^a^
*Staphylococcus aureus*	0.75–1.5	1.5–3	6–12^a^	12–24^a^
Gram-negative bacteria				
*Escherichia coli*	3–6	3–6	3–6^a^	6–12^a^
*Vibrio alginolyticus*	6–12	12–24	6–12	24–48
*Vibrio harveyi*	3–6	6–12	6–12	12–24
*Vibrio parahaemolyticus*	3–6	6–12	12–24	12–24
*Edwardsiella tarda*	0.75–1.5	1.5–3	<0.375	3–6
*Aeromonas hydrophila*	0.75–1.5	0.75–1.5	>96^a^	>96^a^
*Pseudomonas fluorescens*	1.5–3	1.5–3	1.5–3^a^	3–6^a^
*Pseudomonas aeruginosa*	12–24	24–48	>96	>96
Yeast				
*Pichia pastoris GS115*	3–6	12–24	3–6	3–6
*Candida albicans*	12–24	24–48	24–48^a^	24–48^a^
Filamentous fungi				
*Aspergillus niger*	6–12	6–12	48–96^a^	48–96^a^
*Fusarium graminearum*	6–12	6–12	ND	ND
*Fusarium solani*	6–12	12–24	ND	ND

[1]. ND represents not done.^a^ Data comes from

### 2.4 Antimicrobial assay

The antimicrobial activity of the synthetic peptide against selected microorganisms was determined using a broth microdilution assay. The minimum inhibitory concentration (MIC) and minimum bactericidal concentration (MBC) were determined as previously described [1]. For each series of experiments, both blank (no peptides) and negative (no bacteria) controls were included.

### 2.5 Antiparasitic assay

In 2008, the Department of Agriculture of the People's Republic of China classified marine white spot disease as a Class II animal epidemic [26]. *C. irritans*, one of the most devastating marine fish parasites, is the pathogen of marine white spot disease [1], [2]. *C. irritans* has four lifecycle stages, and the parasitic stage (trophont) feeds on the epithelial layers of the host [27]–[29]. Thus, this parasite has intimate contact with the host, causing severe damage to the host [28], [29].

*C. irritans* was isolated, identified and cultured as previously described [1]. Then, the *C. irritans* trophonts were collected from the gills, skin and pterygiophore of heavily infected *Sparus latus* on the third day after *C. irritans* infection. After being rinsed thoroughly with sterile-filtered seawater, active trophonts were cultured in 24-well tissue culture plates with sterile-filtered seawater at 25–26°C and were used for the antiparasitic assay. The antiparasitic assay was performed as previously reported [1]. Briefly, 100 µl of parasite suspension containing 3 trophonts was added to a 96-well tissue culture plates. Then, 11 µl of Pc-pis-His (6–48 µM final concentration), sea water (blank control) or diluent I (negative control) was added to the 100 µl suspensions of trophonts. At different time points after incubation at 25–26°C, motility and changes in the morphology of trophonts were assessed under an inverted microscope.

### 2.6 The stability assay

#### 2.6.1 Effects of salinity

The effect of salinity on the antimicrobial activity of Pc-pis and Pc-pis-His was investigated based on the antimicrobial assay previously described [1], with slight modifications in the broth. Logarithmic phase *Staphylococcus aureus* was incubated in modified broth with varying concentrations of NaCl (0–2560 mM), MgCl_2_ (0–160 mM), or CaCl_2_ (0–40 mM). After incubation for 24 h at 28°C, bacteria growth was examined. The MIC was defined using the same criteria as the antimicrobial assay previously described [1].

#### 2.6.2 Acid- and alkali-tolerance

Acid- and alkali-tolerance of Pc-pis and Pc-pis-His was investigated in an antimicrobial assay as previously described [1]. The only difference was that the MIC was determined against *S. aureus* in a modified MHB with varying pH (4.5–10.5).

#### 2.6.3 Heat stability

The effect of heat treatment on the antimicrobial activity of Pc-pis and Pc-pis-His was investigated with an antimicrobial assay as previously described [1]. However, before either peptide was used in the assay, it was boiled for 0–60 min. After a 24 h incubation at 28°C, the MIC was determined by examining bacteria growth.

### 2.7 Hemolytic activity

Hemolytic activity was determined as previously reported [7], [8], [10], with the following modification; 135 µl of 5% (v/v) erythrocyte suspension from healthy humans in PBS (phosphate buffer solution) was incubated with 15 µl of the serial dilutions of Pc-pis and Pc-pis-His (0.75–48 µM final concentration), 0.9% normal saline (blank control) or 0.1% (v/v) SDS (positive control). After a 30 min incubation at 37°C, all samples were centrifuged for 10 min at 5000 rpm at room temperature. The supernatant (120 µl) was transferred to a 96-well tissue culture plate, and the absorbance at 405 nm was measured for each solution. The percentage of hemolysis was calculated using the following equation: Hemolysis (%)  = OD_405 nm_ sample/OD_405 nm_ positive control. All experiments were conducted in accordance with the guidelines approved by the Institutional Animal Care and Use Committee at the Xiamen University.

### 2.8 Statistical analysis

All tests were performed in duplicate and replicated at least three times. For the antiparasitic assay and hemolytic activity, the data were expressed as the means ± SD (standard deviation), and the SDs of the means of each set were represented on each graph. The significant difference (*P*<0.05) and extremely significant difference (*P*<0.01) between the Pc-pis-His group and the Pc-pis group at each concentration was determined using one-way ANOVA with Tukey post-hoc test.

## Results

### 3.1 Peptide molecular characterization

**Figure 1:**
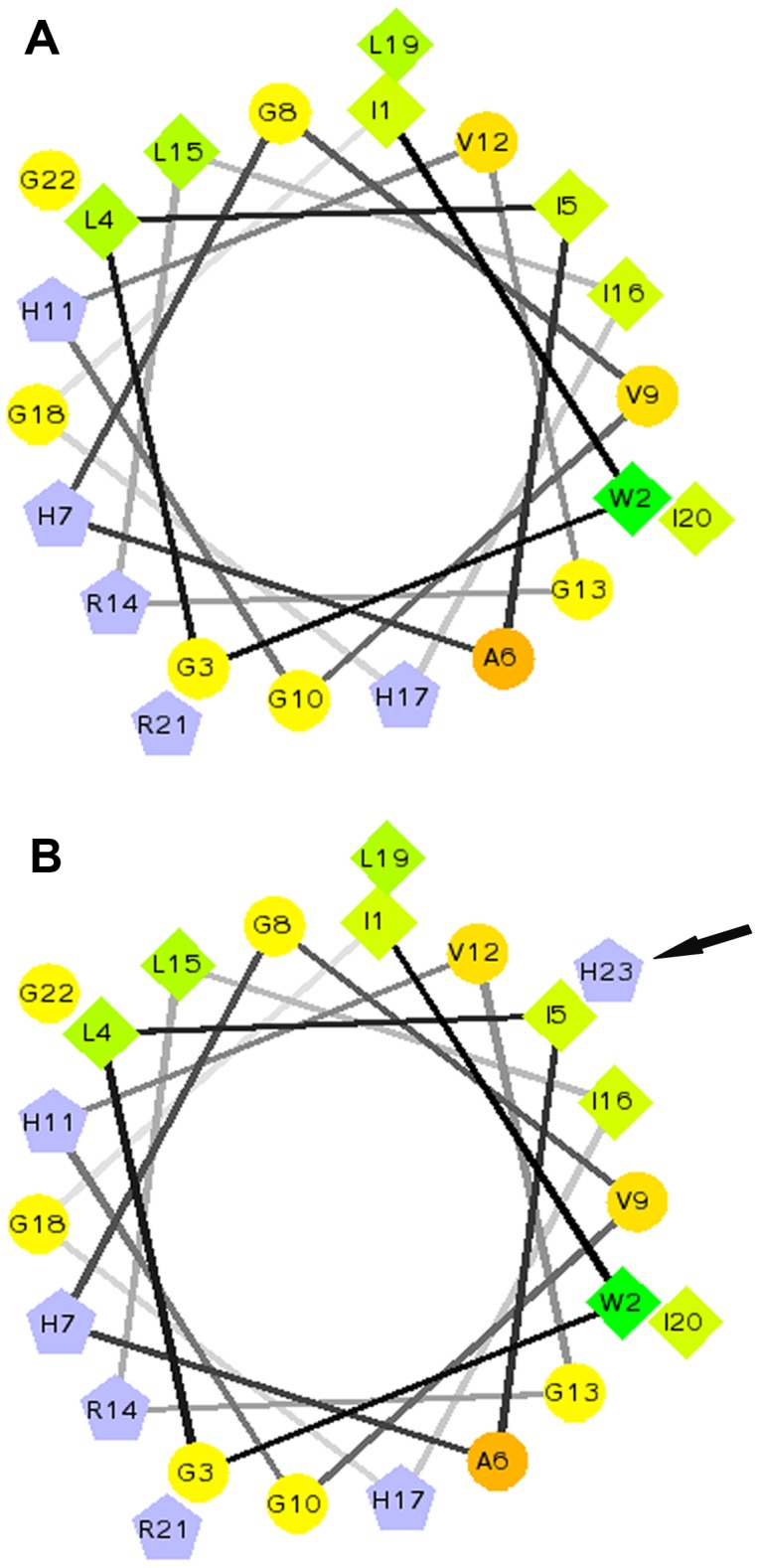
The Schiffer-Edmundson helical wheel diagrams of Pc-pis (A) and Pc-pis-His (B). The amino acid residues are consecutively numbered starting from the N-terminal of the mature peptides, with lines connecting each consecutive amino acid in the sequence. Pentagons and diamonds represent hydrophilic charged and hydrophobic residues, respectively. The circles denote other neutral or polar amino acids. The arrow shows the predicted position of the added histidine residue is in the middle of the hydrophobic phase. Picture A comes from [1].

**Figure 2:**
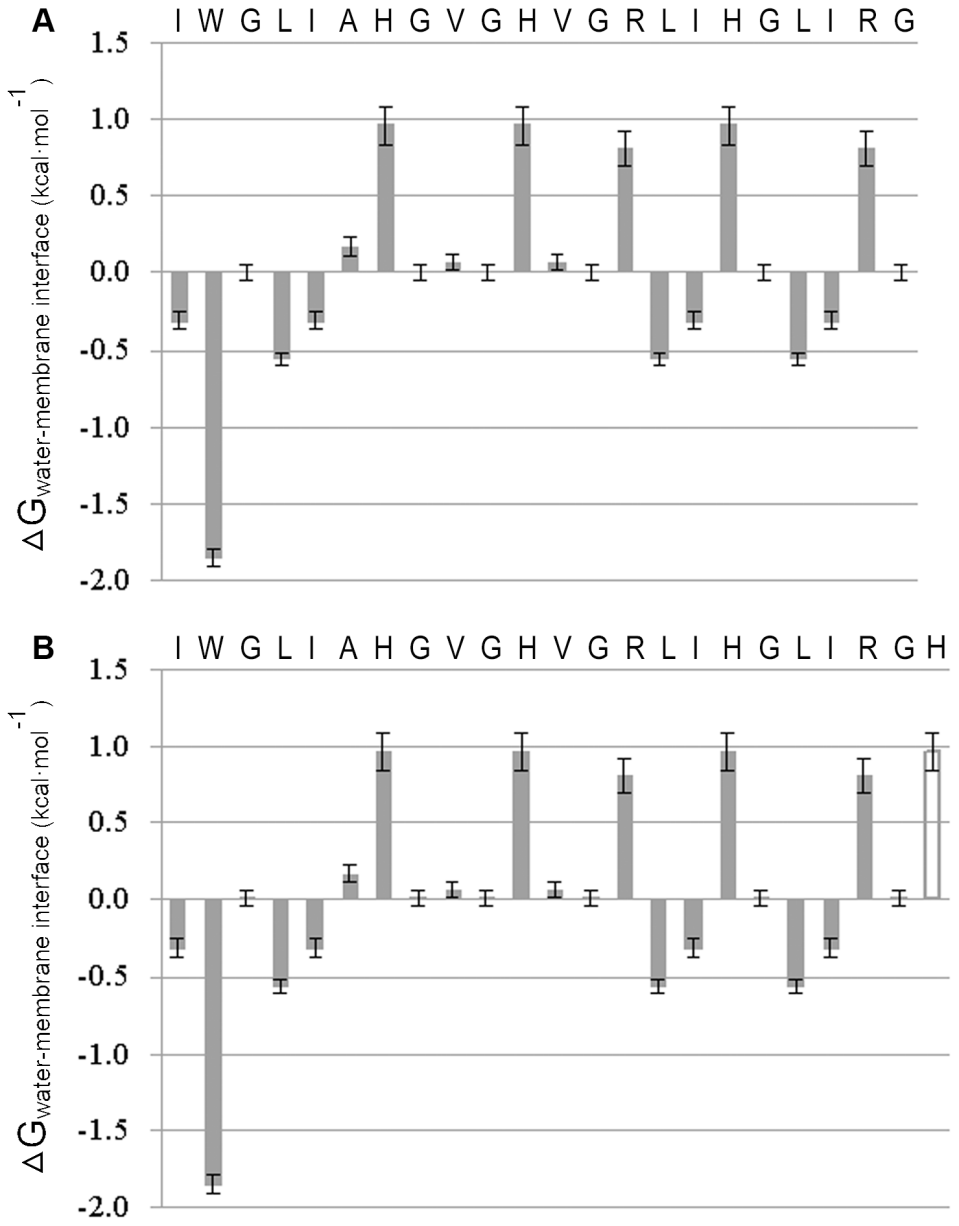
Theoretical prediction of Pc-pis (A) and Pc-pis-His (B) membrane-binding affinity. Based on the whole-residue interfacial hydrophobicity scale as described in [24], the free energies of transfer of amino acids from the water to the palmitoyloleoylphosphatidylcholine (POPC) interface (△G_water-membrane interface_) are represented. The amino acids residues with △G_water-membrane interface_<0 possess lower affinity for the aqueous environment than for the membrane interface region. Compared to Pc-pis, Pc-pis-His has an additional histidine residue (△G_water-membrane interface_ = 0.96±0.12 kcal·mol^−1^, white bars) at the carboxyl terminal, reducing its membrane-binding affinity.

In the study, the Pc-pis analogue, Pc-pis-His, was designed by adding a histidine residue at the carboxyl terminal. The Schiffer-Edmundson helical wheel diagrams showed that Pc-pis (Figure 1A) had a relatively narrow hydrophilic sector and a relatively large hydrophobic phase [1]. The added histidine residue was located in the middle of the hydrophobic phase (Figure 1B), which tends to disrupt the amphipathic α-helix and lowers its amphipathicity. The α-helical structure of Pc-pis-His at the C-terminus predicted using PredictProtein was formed from histidine11 to isoleucine20 (Helix = 43.48%), which was longer than that in Pc-pis (from histidine11 to glycine18, Helix = 36.36%). In addition, the instability index of Pc-pis and Pc-pis-His was −14.42 and −13.36, respectively, which classified the two peptides as stable. Analysis of the amino acid sequences showed that Pc-pis-His (GRAVY = 0.630, charge = 6 and pI = 12.00) had a lower grand average of hydropathicity index (GRAVY), a higher charge and an equal theoretical pI compared to Pc-pis (GRAVY = 0.805, charge = 5 and pI = 12.00). According to the Wimley and White's amino acid interfacial hydrophobicity scale, which predicts the partition of the whole residue from water to the bilayer interfaces [24], [25], the putative membrane-binding affinity followed the trend: Pc-pis > Pc-pis-His (Figure 2).

### 3.2 Antimicrobial spectrum

The antimicrobial activity of the peptides was determined against a representative set of microorganisms (Table 1). The results showed that the synthetic Pc-pis-His exhibited strong antimicrobial activity against all microorganisms tested including primary fish pathogens such as *A. hydrophila*, *Vibrio harveyi*, *Vibrio alginolyticus* and *Vibrio parahaemolyticus* and human bacteria such as *Pseudomonas aeruginosa*, *Escherichia coli* and *Pseudomonas fluorescens*. Compared to Pc-pis with its selective antimicrobial activity, Pc-pis-His showed a relatively more broad-spectrum and more potent antimicrobial activity against the variety of bacterium tested. Especially, Pc-pis-His exhibited a dramatic increase in antimicrobial activity against *A. hydrophila* and *P. aeruginosa* compared to Pc-pis, which made up for the disadvantage of Pc-pis being inactive against *A. hydrophila* and *P. aeruginosa* at concentrations over 96 µM. For all microorganisms tested, the MBC was either equal to or twice MIC, with the exceptions of Pc-pis-His against *Pichia pastoris GS115* and Pc-pis against *V. alginolyticus*.

### 3.3 Antiparasitic assay

**Figure 3:**
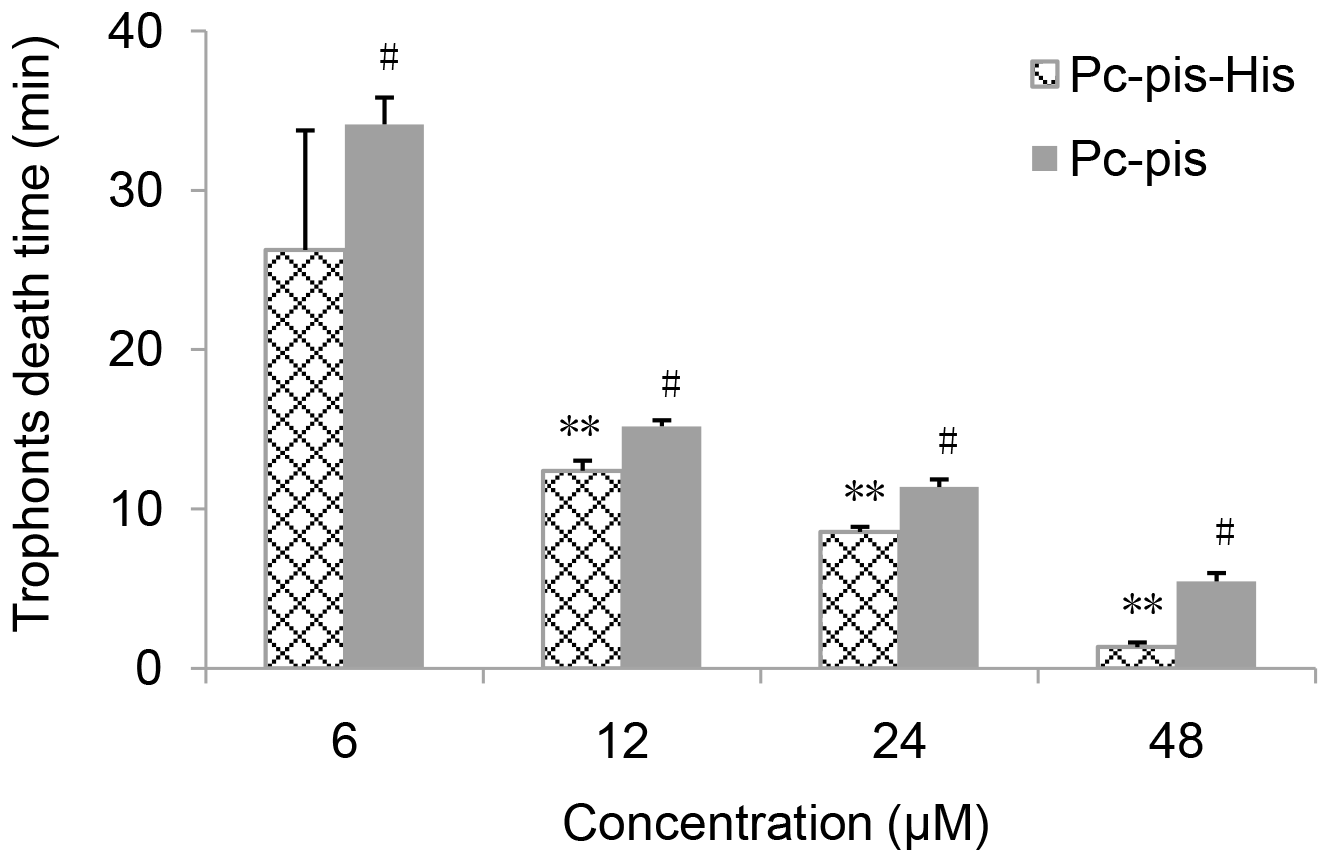
Antiparasitic activity of Pc-pis-His and Pc-pis against *C. irritans* trophonts. The data are shown as the means ± SD (N≥3). Significant differences and extremely significant difference between the Pc-pis-His group and the Pc-pis group at each concentration are indicated with * (*P*<0.05) and ** (*P*<0.01), respectively. # Data comes from [1].

We also explored the antiparasitic activity of Pc-pis-His against *C. irritans* trophonts. As shown in Figure 3, Pc-pis-His was lethal to *C. irritans* trophonts at the concentrations tested. At 48 µM, all trophonts were completely lysed within 1.5 min, and even lower doses, such as 6 µM, killed all trophonts after approximately 30 minutes of exposure. In addition, the rate of trophont death and cytoplasmic leakage was distinctly dose-dependent.

Pc-pis and Pc-pis-His had very similar sequences, differing only in the histidine residue added at the carboxyl terminal, yet the antiparasitic activity of Pc-pis-His was improved significantly at a concentration of 12, 24 and 48 µM. At the concentration of 6 µM, Pc-pis-His, though not significant, also exhibited stronger activity against *C. irritans* trophonts than Pc-pis. Overall, Pc-pis-His demonstrated relatively more potent antiparasitic activity against *C. irritans* trophonts than Pc-pis.

### 3.4 The stability assay

#### 3.4.1 Effects of salinity

We also detected the impact of salinity, which might disrupt the electrostatic interaction between the negatively charged microbial surface and the positively charged Pc-pis and Pc-pis-His. The MIC values against *S. aureus* are shown in Table 2. The MIC values of Pc-pis-His were unchanged in the presence of up to 320 mM NaCl, 160 mM MgCl_2_ and 5 mM CaCl_2_ and only doubled in the presence of 640–2560 mM NaCl and 10–40 mM CaCl_2_, which showed that the salt-tolerance of the peptide was high. By contrast, Pc-pis was relatively more sensitive to salinity, especially to divalent cations. The antimicrobial activity of Pc-pis against *S. aureus* was partially inhibited in the presence of 0–160 mM NaCl and completely inhibited at the concentrations tested (0.75–48 µM) in the presence of 320–2560 mM NaCl, 1.25–40 mM MgCl_2_ and 1.25–40 mM CaCl_2_. Therefore, the added histidine residue at the carboxyl terminal significantly enhanced the antimicrobial activity of piscidins in environments rich in divalent cations.

**Table 2:** Effect of monovalent and divalent cations on activity of Pc-pis-His and Pc-pis against *S. aureus*.

Salt	Concentration (mM)	Pc-pis-His MIC (µM)	Pc-pis MIC (µM)
NaCl	0	0.75–1.5	12–24
	20	0.75–1.5	24–48
	40	0.75–1.5	24–48
	80	0.75–1.5	24–48
	160	0.75–1.5	≥48
	320	0.75–1.5	>48
	640	1.5–3.0	>48
	2560	1.5–3.0	>48
MgCl_2_	1.25	0.75–1.5	>48
	40	0.75–1.5	>48
	80	0.75–1.5	>48
	160	0.75–1.5	>48
CaCl_2_	1.25	0.75–1.5	>48
	5	0.75–1.5	>48
	10	1.5–3.0	>48
	40	1.5–3.0	>48

#### 3.4.2 Acid- and alkali-tolerance

As shown in Table 3, extremely acidic and alkaline environments could result in a partial or complete loss of the bactericidal activity of Pc-pis-His and Pc-pis. A 2-fold decrease in the antimicrobial activity of Pc-pis was observed in acidic environments, and alkaline environments (pH 7.5–9.5) could inhibit completely the antimicrobial activity of the peptide at the concentrations tested (0.75–48 µM). When compared to Pc-pis, Pc-pis-His showed much better acid- and alkali-tolerance. Only a 2-fold increase in the MIC values of Pc-pis-His against *S. aureus* was discovered up to pH 10.5. However, no visible *S. aureus* growth was found in the experimental groups and blank controls below pH 5.5, which implied that the extremely acidic environment rather than Pc-pis-His and Pc-pis inhibited *S. aureus* growth. These results showed that the optimal environmental pH was 5.5–10.5 and 5.5–7.3 for Pc-pis-His and Pc-pis, respectively, which indicated that the added histidine residue at the carboxyl terminal significantly enhanced the antimicrobial activity of piscidins in alkaline environments.

**Table 3:** Effect of acid and alkali on activity of Pc-pis-His and Pc-pis against *S. aureus*.

pH	MIC (µM)	
	Pc-pis-His	Pc-pis
4.5–5.5	—	—
5.5–6.5	0.75–1.5	12–24
6.5–7.0	0.75–1.5	12–24
7.3	0.75–1.5	6–12
7.5	0.75–1.5	>48
8.5	0.75–1.5	>48
9.5	0.75–1.5	>48
10.5	1.50–3	24–48

“—” represents no visible microbe is detected at both blank control group (no peptides) and experimental group.

#### 3.4.3 Heat stability

In addition to salinity and pH, there were other factors, such as temperature, which might affect the activity of AMPs. Table 4 shows that the antimicrobial activity of Pc-pis-His was relatively more susceptible to temperature than that of Pc-pis. When Pc-pis always remained constant in its antimicrobial activity, the antimicrobial activity of Pc-pis-His weakened somewhat with the extension of heat treatment time. However, Pc-pis-His was still effective against *S. aureus* after heat treatment, and the MIC values increased only 2-fold relative to the untreated peptides after boiling for 40–60 min. All data suggested that Pc-pis-His and Pc-pis both had high heat stability.

**Table 4:** Effect of heat treatment on activity of Pc-pis-His and Pc-pis against *S. aureus*.

Boiling time (min)	MIC (µM)	
	Pc-pis-His	Pc-pis
0	0.75–1.5	6–12
10	0.75–1.5	6–12
20	0.75–1.5	6–12
30	0.75–1.5	6–12
40	1.5–3	6–12
50	1.5–3	6–12
60	1.5–3	6–12

### 3.5 Hemolytic activity

**Figure 4:**
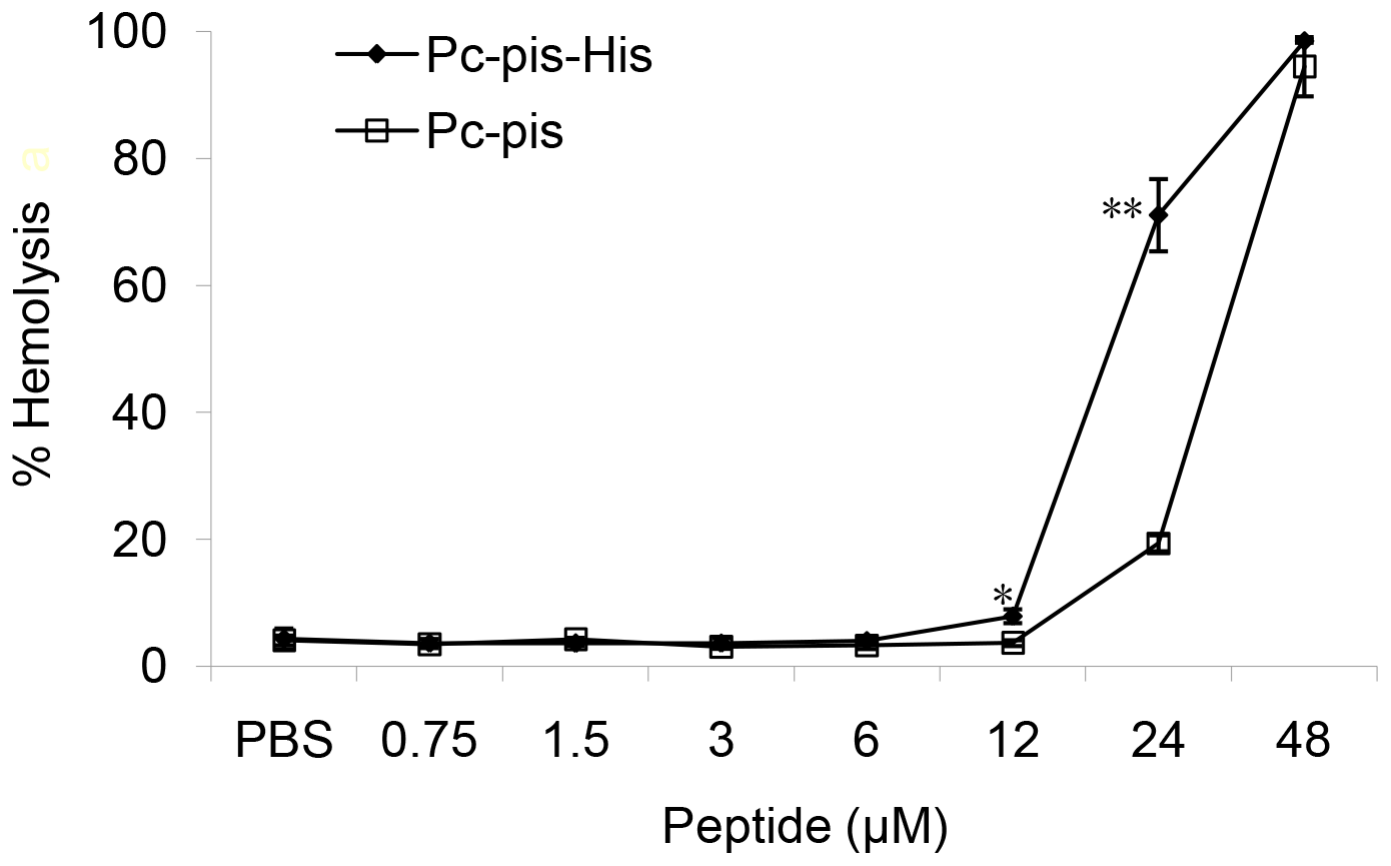
Dose-response of hemolytic activity of Pc-pis-His and Pc-pis against human erythrocytes. The data are expressed as the means ± SD (N≥3). An asterisk (*) and two asterisks (**) represent a significant difference (*P*<0.05) and an extremely significant difference (*P*<0.01) between the Pc-pis-His group and the Pc-pis group at each concentration, respectively.

To investigate the cytotoxicity of Pc-pis and Pc-pis-His, the ability of the peptides to cause the lysis of human erythrocytes was measured. As shown in Figure 4, the hemolytic activity of the peptides was significantly dependent on the dose of the peptides. The Pc-pis was not hemolytic against 5% human erythrocytes at 12 µM, and 15.303% hemolysis was demonstrated by the peptide at a concentration of up to 24 µM, compared to blank control. Compared to Pc-pis, the hemolytic activity of Pc-pis-His was enhanced significantly at 12 and 24 µM. Nevertheless, the Pc-pis-His was not hemolytic at up to 6 µM, an active concentration against most microorganisms tested, and only elicited 3.573% hemolysis up to 12 µM, compared with blank control.

## Discussion

### 4.1 Structure characterization

In this study, to probe how structure, biological activity and the stability of piscidins are affected by a histidine residue and to provide a reasonable basis for the development of potential therapeutic agents against parasitic and microbial infections, the Pc-pis analogue, Pc-pis-His, was designed by adding a histidine residue at the carboxyl terminal. Although there was only a simple difference in an amino acid between Pc-pis-His and Pc-pis, subtle structural changes were observed. Pc-pis-His had more positive charges (20% increment) under physiological conditions, longer α-helical content (7.12% increment), lower amphipathicity (Figure 1), less hydrophobicity (27.8% reduction) and lower membrane-binding affinity (Figure 2) than Pc-pis. In addition, the imidazolyl of the added histidine residue might also provide a better hydrogen bonding property between the peptides and microbial membranes to Pc-pis-His and stronger electrostatic cation-π interaction for peptide self-association within membranes [19], [20], [30]. To date, the structural properties of many AMPs, such as piscidin 1 [10], [23], cathelicidin [9] and BP100 [12], are also changed with the incorporation of some amino acids, and the changes greatly influence the biological activity of the peptides. Thus, the changes in the various structural biology characteristics that are endowed through the added histidine residue might also affect the biological activity of the peptide.

### 4.2 Biological activity analysis

To develop therapeutic drugs with therapeutic potential, the developed AMPs are supposed to be toxic to pathogen cells but should be non-toxic or have low toxicity in eukaryotic cells. The biological activity analysis revealed that Pc-pis-His exhibited certain activity against all bacteria tested as well as *C. irritans* trophonts and no hemolysis against human erythrocytes at up to 6 µM, a concentration highly active against most of the microorganisms tested. Pc-pis-His showed a relatively more broad-spectrum and more effective antimicrobial activity, relatively stronger antiparasitic activity and slightly enhanced hemolytic activity compared with Pc-pis. The positively charged histidine residue added at the carboxyl terminal of Pc-pis might be responsible for the increased antimicrobial, antiparasitic and hemolytic activity of Pc-pis-His. Similarly, hybrid striped bass Pis-1 PG also displayed more potent antimicrobial activity than Pis-1 PG-2HA due to the substitutions of two alanines in the N-terminus of Pis-1 PG-2HA with two histidines [10], and similar increased hemolytic activity was also observed in R-BP100 that had the lysines replaced with arginines compared with its parent analogue BP100 [12].

The importance of histidine residues on the biological activity of piscidins was further supported in this study, which mainly manifested in the following several aspects: firstly, the initial electrostatic interaction between the cationic AMPs and the negatively charged lipid head groups of membranes was enhanced due to the fact Pc-pis-His possessed relatively more positive charges than Pc-pis [10], [23]. The electrostatic interaction was decisive for the activity of cationic AMPs against various pathogens, especially for antimicrobial activity [12], [31]. Secondly, the increased α-helical content, which influence the hydrophobic peptide-membrane interactions in the binding process, might have been responsible for the relatively effective activity of Pc-pis-His, especially for its increased antiparasitic activity and enhanced hemolytic activity [9], [10], [31]–[34]. Finally, compared to Pc-pis, the relatively better hydrogen bonding property and stronger electrostatic cation-π interaction of Pc-pis-His might more effectively facilitate its interaction with the target membrane surfaces and inner hydrophobic environment, especially for gram-positive bacteria [12], [19], [20]. Nevertheless, the slightly reduced facial amphipathicity, overall hydrophobicity and membrane-binding affinity, being often correlated with reduced hydrophobic interactions with membrane interfaces, did not decrease the antimicrobial activity and hemolytic activity of Pc-pis-His [6], [13], [31], [35]. For this apparent contradiction, one possible explanation was that a balanced relationship among various structural biology characteristics might be demanded in the antimicrobial, antiparasitic and hemolytic mechanisms employed by the two peptides. Moreover, these results are another reminder of how complex peptide-membrane interactions can be.

In this study, an interesting phenomenon was observed from the results of biological activity. Human erythrocytes were largely resistant, protozoan parasite *C. irritans* trophonts exhibited an intermediate degree of sensitivity and bacteria were most susceptible to both Pc-pis-His and Pc-pis. The activity of piscidin 4 was similar and demonstrated the most potent antimicrobial activity and a relatively high potency for the protozoan parasite *Ichthyophthirius multifiliis* trophonts compared to its negligible toxicity for erythrocytes [16], [36]. The differential activities might be due to the differences in the composition of the bacteria, protozoan and erythrocyte membranes [36], [37]. The sterol existing in the human erythrocyte outer leaflet but rarely in protozoan membranes and never in bacteria membranes confers more ordered membrane acyl chains and a more rigid and stabilized bilayer structure to erythrocyte membranes [36]–[40], which might inhibit the AMPs from permeating deeply into the erythrocyte membranes.

### 4.3 The stability

The AMPs that really gather both academic and industrial interest are the peptides with not only potent activity against pathogen cells and low toxicity towards eukaryotic cells but also stable activity. Whether the antimicrobial activity of the peptide would be affected by the various interference factors play a decisive part in application value. In the present study, the effects of high temperature, salinity and pH on antimicrobial activity of Pc-pis and Pc-pis-His were comparatively evaluated to further investigate the contribution of a histidine residue to antimicrobial performance.

The antiparasitic activity and the stability assay indicated that Pc-pis-His possessed relatively more optimistic salt-, acid- and alkali-tolerance than Pc-pis, especially in the divalent cation and alkaline environments. The tolerance of the two analogues was in agreement with their charges and hydrogen bonding properties, both of which are closely related to AMP antimicrobial activity [12], [19], [20], . In the presence of high concentration salinity, acids and alkalis, the electrostatic interaction and hydrogen bonding property of the peptide-membrane might be competing between the cations (H^+^, Na^+^, Mg^++^, Ca^++^) and the cationic polypeptides or between the alkali ion (OH^–^) and the negatively charged microbial membranes [8], [12], [19], [20], [30], [33]. The increased positive charges and hydrogen bonding property conferred higher competitiveness to Pc-pis-His than Pc-pis regarding the interaction of its peptide-membrane. However, the competitive electrostatic interactions and hydrogen bonding resulted in a partial or complete loss of Pc-pis-His and Pc-pis antimicrobial activity. In general, the strategically placed histidine residue renders piscidins with relatively stable activity in high concentration salinity, acidic and alkaline environments.

According to the heat stability study, both Pc-pis-His and Pc-pis were still effective against *S. aureus* after the peptides were boiled for 10–60 min, which showed that Pc-pis-His and Pc-pis both had a good heat stability for applications. That might be due to special structure of piscidins that is linear and mainly unstructured in water [16], [33], [36]. However, the activity of Pc-pis-His was relatively susceptible to temperature compared with Pc-pis. It was likely that the additional histidine residue had an imidazolyl side chain, which resulted in Pc-pis-His being affected more easily by temperature [19], [20], although more research is needed for clarification.

When considered in conjunction with the effects of salinity, pH and temperature on antimicrobial activity, the added histidine residue at the carboxyl terminal of Pc-pis conferred desired heat-tolerance and better salt-, acid- and alkali-tolerance to Pc-pis-His than in Pc-pis, especially in the divalent cation and alkaline environments. Thus, the antimicrobial activity of Pc-pis-His was less disturbed by various interference factors in the application process, which indicated that Pc-pis-His was obviously better suited to be put into production than Pc-pis. All data in this report also indicated that histidine residues may be especially useful amino acids for designing piscidins analogues intended to function within divalent cation and alkaline environments.

## Conclusions

In this study, a new AMP, Pc-pis-His, was designed based on the amino acid sequence of its parent analogue Pc-pis. This paper is the first report that the antiparasitic and antimicrobial activity of piscidins could be enhanced significantly through the introduction of a histidine residue at the carboxyl terminal. The added histidine residue also conferred desired heat-tolerance and better salt-, acid- and alkali-tolerance to piscidins, especially in divalent cation and alkaline environments. Overall, the results obtained in this study provide a reasonable basis for designing novel piscidins that have potent, broad-spectrum and stable antimicrobial activity and also provide new insight into the future development of piscidins as potential therapeutic agents against microbial and external protozoan parasite infections.
